# Identifying relevant topics and training methods for emergency department flow training

**DOI:** 10.1007/s43678-022-00390-1

**Published:** 2022-10-15

**Authors:** Christina Young, Christopher Patey, Paul Norman, Teresa Chan, Oliver Hurley, Michelle Swab, Shabnam Asghari

**Affiliations:** 1 Centre for Rural Health Studies, Discipline of Family Medicine, Faculty of Medicine, Memorial University of Newfoundland, St. John’s, NL Canada; 2 Carbonear General Hospital, Carbonear, NL Canada; 3 Faculty of Health Sciences, McMaster University, Hamilton, ON Canada; 4 Health Sciences Library, Memorial University of Newfoundland, St. John’s, NL Canada

**Keywords:** Training, Emergency department staff, Flow, Wait-times, Formation, Personnel des services d'urgence, Flux, Temps d'attente

## Abstract

**Purpose:**

Despite the importance of patient flow to emergency department (ED) management, there is a need to strengthen and expand training in flow strategies for practicing ED staff. To date, there has been limited academic inquiry into the skills and training that ED staff require to improve patient flow. As part of a quality improvement initiative, our team aimed to identify the topics and training methods that should be included in flow training for ED staff.

**Methods:**

We conducted an integrative review and modified Delphi. For the integrative review, we sought to identify appropriate skills, training strategies, and training modalities to include in a curriculum for ED staff. The findings from the review were compiled and distributed to Canadian experts in ED efficiency through a modified Delphi, including physicians, nurses, and nurse practitioners.

**Results:**

Our literature search retrieved 8359 articles, of which 46 were included in the review. We identified 19 skills, 9 training strategies, and 12 training modalities used to improve ED efficiency in the literature. For the modified Delphi, we received responses from 39 participants in round one and 28 in round two, with response rates of 57% and 41%, respectively. The topics chosen by the most respondents were: “flow decisions,” “teamwork,” “backlog and surge management,” “leadership,” and “situational awareness.”

**Conclusion:**

Our findings suggest that flow training should teach ED staff how to make decisions that improve flow, work more effectively as a team, manage patient backlog and surge, improve leadership skills, and develop situational awareness. These findings add to a gap in the academic literature regarding the training ED staff require to improve patient flow.

**Supplementary Information:**

The online version contains supplementary material available at 10.1007/s43678-022-00390-1.

## Résumé

**Objectifs:**

Malgré l'importance du flux des patients vers la gestion des services d'urgence (SU), il est nécessaire de renforcer et d'étendre la formation sur les stratégies de flux pour le personnel en exercice des SU. Jusqu'à présent, peu d'études universitaires ont été menées sur les compétences et la formation dont le personnel des urgences a besoin pour améliorer le flux des patients. Dans le cadre d'une initiative d'amélioration de la qualité, notre équipe a cherché à identifier les sujets et les méthodes de formation qui devraient être inclus dans la formation sur les flux pour le personnel des urgences.

**Méthodes:**

Nous avons procédé à une étude intégrative et à une analyse Delphi modifiée. Dans le cadre de l'étude intégrative, nous avons cherché à déterminer les compétences, les stratégies de formation et les modalités de formation appropriées à inclure dans un programme de formation destiné au personnel des services d'urgence. Les conclusions de l'étude ont été compilées et distribuées à des experts canadiens en matière d'efficacité des urgences par le biais d'un Delphi modifié, comprenant des médecins, des infirmières et des infirmières praticiennes.

**Résultats:**

Notre recherche littéraire a permis de récupérer 8 359 articles, dont 46 ont été inclus dans l'examen. Nous avons identifié 19 compétences, 9 stratégies de formation et 12 modalités de formation utilisées pour améliorer l'efficacité de l'urgence dans la littérature. Pour le Delphi modifié, nous avons reçu des réponses de 39 participants au premier tour et de 28 au deuxième tour, avec des taux de réponse de 57 % et 41 %, respectivement. Les sujets choisis par le plus grand nombre de répondants sont : " décisions de flux ", " travail d'équipe ", " gestion de l'arriéré et de l'afflux ", " leadership " et " conscience situationnelle ".

**Conclusions:**

Nos résultats suggèrent que la formation sur le flux devrait enseigner au personnel des urgences comment prendre des décisions qui améliorent le flux, travailler plus efficacement en équipe, gérer l'arriéré et l'afflux de patients, améliorer les compétences en leadership et développer la conscience de la situation. Ces résultats viennent combler une lacune dans la littérature académique concernant la formation dont le personnel des urgences a besoin pour améliorer le flux des patients.

## Clinician’s capsule

**Table:** 

***What is known about the topic?*** Despite the importance of patient flow to emergency department (ED) management, there is a need to improve training in flow strategies for practicing emergency department staff
***What did this study ask?*** What topics and training methods should be used in a curriculum for emergency department staff to improve patient flow?
***What did this study find?*** The topics chosen by the most respondents were: “flow decisions,” “teamwork,” “backlog and surge management,” “leadership,” and “situational awareness.”
***Why does this study matter to clinicians?*** Teaching ED staff how to improve flow is critical to decreasing wait times for patients seeking emergency care

## Introduction

Canadian emergency departments (EDs) suffer from overcrowding, long wait times, and low levels of patient satisfaction [1]. Excessive wait times can lead to increased morbidity and mortality, untreated pain and discomfort, and increased violence or aggression against staff [2]. They can also prevent access to urgent and required care [3]. While many issues contribute to this crisis, poor patient flow through the ED is a contributing factor [4].

Despite the importance of patient flow to ED management, there is a need to strengthen and expand training in flow strategies for practicing ED staff. The *Standards of Accreditation for Residency Programs in Emergency Medicine* from the Royal College of Physicians and Surgeons of Canada states that residents transitioning to practice should regularly be responsible for the “clinical management of the emergency department and of overall patient flow” [5]. However, this directive does not outline how these skills should be taught. Patient flow is also not included as a priority topic for residents completing training in emergency medicine through the College of Family Physicians of Canada [6]. Physicians already in practice may not have received this training during a clinical residency or might require a refresher course to strengthen their existing knowledge [7]. Additionally, charge nurses, who are often responsible for managing patient flow, typically receive no specialized training in this area, instead learning these skills through observation and experience [8].

In addition to the gap in flow training for ED staff, limited academic literature outlines the specific concepts and strategies that should be employed in ED flow training. Existing literature has begun to address this gap by identifying and describing strategies for teaching ED flow, primarily to medical residents [9–14]. However, there is a need to identify a comprehensive list of topics and training methods for flow training for ED staff, including emergency physicians, nurse practitioners, nurses, and medical learners.

As part of a quality improvement initiative aimed at improving patient flow and decreasing ED wait times [15], our team sought to identify the topics that should be included in a flow training course for ED staff. We aimed to address the following two questions: (1) What skills do ED staff require to improve patient flow? (2) What training strategies and modalities are effective in helping ED staff develop these skills?^1^ We conducted an integrative review and modified Delphi to answer these questions. Our findings address steps one and two of Kern’s six-step approach to curriculum development [16].

## Methods

We conducted an integrative review [17] to identify appropriate skills, training strategies, and training modalities to include in a curriculum for ED staff. The findings from the review were compiled and distributed to Canadian experts in ED practice through a modified Delphi.

### Integrative review

Our team conducted an integrative review to identify potential topics to include in a curriculum to improve patient flow, based on Whittemore and Knalf’s [17] methodology for integrative reviews and qualitative content analysis [18]. Integrative systematic reviews combine literature from various methodological sources, including experimental and non-experimental research [17]. We selected this type of review since we aimed to examine the full breadth of skills, training strategies, and training modalities employed to train ED staff to improve flow from various academic sources.

Our research question was developed based on the population, concept, and context framework [19]. We asked which skills and training strategies contribute to improved efficiency among ED staff. A medical librarian searched the online databases *Ovid MEDLINE*, *Embase*, *CINAHL*, and *PsycINFO* on January 27, 2021 (with an updated search on January 28 2022). Search terms were developed by content experts (CP, PN, CY, SA) and a medical librarian and included variations of “emergency department,” “training,” and “wait time(s).” Articles were stored and managed in *Covidence*.

We included peer-reviewed journals, Ph.D. dissertations, and commentaries retrieved by the database search published in English that discuss the skills or training that emergency department staff require to improve patient flow. We excluded conference abstracts, articles published in languages other than English, and studies that did not focus on the emergency department or skills and training related to patient flow.

Two research team members screened each title and abstract, excluding those unrelated to our research objectives. Conflicts were resolved through a discussion between the two reviewers; a third team member provided feedback when necessary. We repeated the same process for the full-text review. We conducted a backward and forward reference search of all included articles to determine if any additional studies should be considered.

At this stage, we developed an extraction tool to aid in organizing and analyzing data from the included articles. The tool gathered the publication information, research question, research design, methods, type of intervention, target group, sample size, findings, and conclusions for each article. Consistent with the methods indicated for integrative reviews, we extracted qualitative data from both quantitative and qualitative studies [17]. The team calibrated the tool by extracting data from five articles and meeting to compare data and make amendments to the tool. Two research team members then extracted relevant data from each remaining article, meeting to resolve any discrepancies.

A research team member with expertise in qualitative research (CY) coded and analyzed the extracted data [17, 18]. Using the qualitative analysis software *QDA Miner,* she coded the extracted data until all data segments were assigned to a category. The initial coding list was refined based on feedback from members of the research team: we combined similar codes or renamed them to reflect the data organized within that code or the terms typically employed in clinical practice. The research team reviewed and approved the final code list, which we used as the foundation for a modified Delphi.

### Modified Delphi

A modified Delphi is a method for gaining a consensus from a panel of experts through a series of structured questionnaires [20]. After completing the integrative review, our team conducted a modified Delphi to obtain expert feedback on which skills, training strategies, and training modalities should be included in our training curriculum. We obtained ethical approval to conduct the Delphi from the Newfoundland and Labrador Health Research Ethics Board (HREB Reference #2019.264).

To conduct the modified Delphi, we designed a survey using *Qualtrics.* Data were divided into training topics, strategies, and modalities. Participants were asked to identify which items from each section should be included in a flow training curriculum and any topics, strategies, or modalities that were absent from our list. Space was also provided for participants to provide additional written comments. We pre-tested the survey with members of the research team and amended it based on their feedback.

We conducted purposeful sampling [21] to recruit a sample representing gender, years of practice, occupation, and the province of residence. We invited nurses, nurse practitioners, and emergency physicians to participate, including authors of studies in the integrative review and others known to have expertise in ED research or practice in Canada. Some participants were also recruited through snowball sampling. We recruited participants via email using a standard script that provided details about the research project and a link to the survey.

For the second round of the Delphi, we designed a survey in *Qualtrics* to ensure that the findings from the first round were comprehensive. We invited all individuals who were contacted to complete the first round. We presented the data using the same categories as round one. This round differed because we provided participants with a complete list of items for each category and asked whether each list was comprehensive and, if not, which additional items should be added to each category.

The findings from the first round of the Delphi were analyzed by counting which topics, training strategies, and training modalities were selected most frequently and comparing the data based on occupation, years of practice, and gender. For the second round, we examined whether any comments added by participants constituted novel concepts. At this stage, we determined that consensus had been achieved and a third round of review was unnecessary.

## Results

### Integrative review

**Fig. 1:**
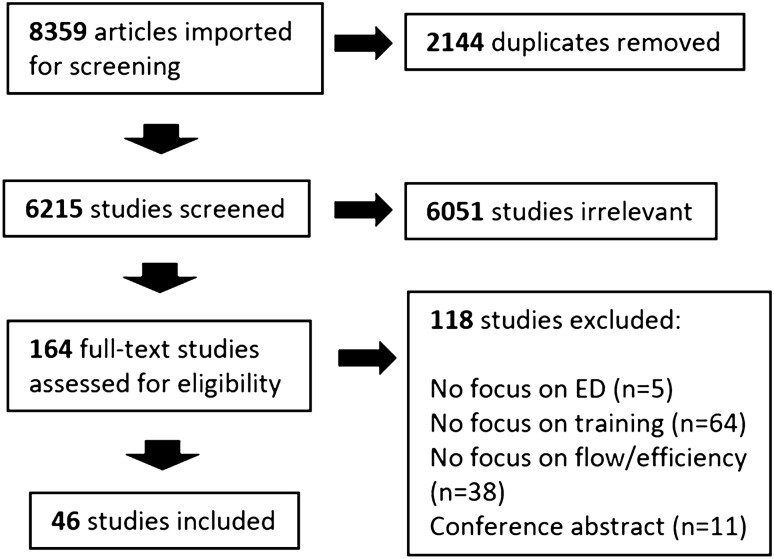
PRISMA for integrative review

**Fig. 2:**
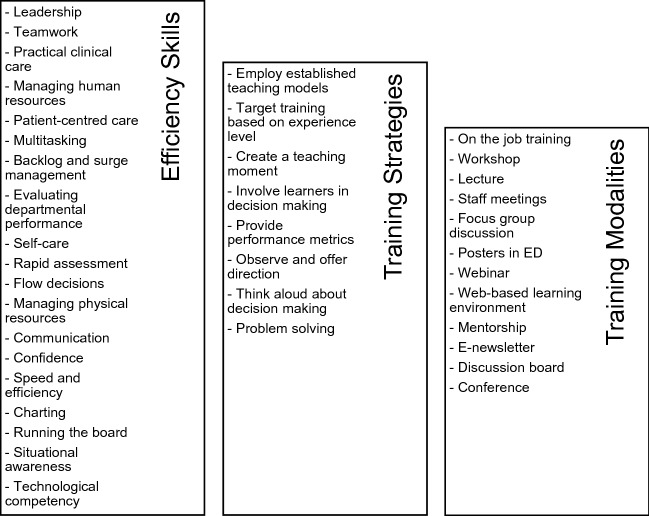
Efficiency skills, training strategies, and training modalities identified in the integrative review

The initial search yielded 8359 results, of which 2144 were duplicates. Of the 6215 articles that underwent title and abstract screening, reviewers deemed 6051 irrelevant (see Fig. 1). After conducting a full-text screening of the remaining 164 articles, 46 were included in the review (see supplement for details about each article). We did not add any new articles through the backward and forward search of included studies. Most studies were conducted in the United States (37%) and Canada (33%), with the remaining originating from Australia (15%) and Europe (15%). Study design included mixed-methods (26%), quantitative (24%), commentary (22%), qualitative (20%), observational (4%), simulation (2%), and review (2%). Physicians were included as a target group in 59% of the articles, followed by nurses (37%), residents (30%), and other ED staff (13%), including nurse practitioners, physician assistants, and medical students. We identified 19 skills, 9 training strategies, and 12 training modalities (see Fig. 2) used to teach ED flow.

### Modified Delphi

We received responses from 39 participants in round one and 28 in round two, with 57% and 41% response rates, respectively. The majority of respondents in round one were emergency physicians (56%), followed by nurse practitioners (21%) and then nurses (15%). Similar ratios were present in the second round (physicians: 68%; nurses: 14%; nurse practitioners: 11%). Both rounds had a greater proportion of responses from men than women (see Table 1).

**Table 1:** Demographic details of modified Delphi participants

	Round one (39 total)	Round two (28 total)
*Occupation*		
Emergency physician	22 (56%)	19 (68%)
Nurse practitioner	8 (21%)	4 (14%)
Nurse	6 (15%)	3 (11%)
Other	3 (8%)	2 (7%)
*Gender*		
Man	22 (56%)	18 (64%)
Woman	17 (44%)	10 (36%)
*Years of practice*		
0–5	5 (13%)	3 (11%)
6–10	6 (15%)	5 (17%)
11–15	8 (21%)	4 (14%)
15 +	20 (53%)	16 (57%)

In round one, each of the topics identified in the integrative review were selected by at least 44% of participants (see Table 2). The topics chosen by the most respondents were as follows: “flow decisions,” “teamwork,” “backlog and surge management,” “leadership,” and “situational awareness.” Participants added two new topics we had not identified through the integrative review: “appropriate limitation of workup” and “admission avoidance.” Similarly, all training strategies were selected by at least 50% of the sample, with the most popular being: “providing clear instruction and feedback,” “thinking aloud about decision making,” “observing learners and offering direction,” “involving learners in decision-making,” and “problem-solving.”

**Table 2:** Number of respondents in round one who selected each topic for inclusion in a flow curriculum

Topics	Number of respondents
Flow decisions	37 (95%)
Teamwork	34 (87%)
Backlog and surge management	33 (85%)
Leadership	31 (79%)
Situational awareness	31 (79%)
Effective supervision of junior staff	31 (79%)
Rapid assessment	30 (77%)
Speed and efficiency	28 (72%)
Communicating with peers	27 (69%)
Patient-centered care	26 (67%)
Managing physical resources	26 (67%)
Evaluating departmental performance	26 (67%)
Running the board	25 (64%)
Charting	23 (59%)
Technological competency	23 (59%)
Managing human resources	23 (59%)
Practical clinical care	22 (56%)
Rapid task shifting	22 (56%)
Confidence	17 (44%)
Self-care	17 (44%)
Other	5 (13%)

In general, the results for training modalities were more uneven. The five most popular modalities—“on-the-job training,” “simulation,” “workshop,” “mentorship,” and “focus group discussion”—were selected by at least 58% of the sample. However, fewer than 25% of respondents chose the bottom five modalities. These included (in descending order) “web-based learning environment,” “webinar,” “conference,” “placing informative posters in the emergency department,” and “e-newsletters.”

In round one, we identified two notable differences among respondents based on occupation. Of the primary care providers surveyed, 81% of emergency physicians selected “speed and efficiency” as relevant to a flow training curriculum, compared to only 38% of nurse practitioners. In addition, while 75% of nurse practitioners and 100% of nurses identified “patient-centred care” as a relevant topic, only 52% of emergency physicians selected this item as applicable to ED flow training.

In round two, 86% of participants indicated that our list of curriculum topics, training strategies, and training modalities was comprehensive. Only four participants (14%) provided suggestions for additional topics. Based on our analysis and input from clinical team members, we determined that only one of these comments constituted a novel concept (“impact of community and hospital resources on ED flow”), while the remaining three could be categorized under existing topics. Four participants (14%) also identified additional training strategies, while two (7%) suggested further training modalities. Our team analyzed these responses and found that they could be captured under existing categories or were unrelated to ED flow training.

## Discussion

### Interpretation of findings

In general, the modified Delphi confirmed the findings from the integrative review. The experts we surveyed appeared to prioritize those skills related to interpersonal conduct among the emergency department staff (e.g. teamwork and leadership) and the management of routine flow and surge management. Both rounds of the modified Delphi had more responses from men than women; however, given that emergency physicians constituted over 55% of the sample in both rounds, this gender composition approximately reflects that found in the study population [22]. We noted that emergency physicians were likelier to identify “speed and efficiency” as an essential topic than nurse practitioners. We also found that nurses and nurse practitioners were more likely to identify “patient-centred care” as a relevant topic than physicians—an underexplored topic in emergency care [23]. These findings may reflect the different responsibilities, training, and ideological perspectives of healthcare professionals practising in the ED. They may also reflect the payment models of each profession. While nurses and nurse practitioners are often paid at a set salary rate regardless of their output, physicians are incentivized to be more productive through fee-for-service funding models.

### Comparison to previous studies

Our findings build on existing literature that describes strategies for teaching ED flow, particularly for medical residents [8–14]. This literature emphasizes the importance of teaching management and leadership skills necessary for improving patient flow and efficiency through constructive feedback, leading by example, and providing opportunities for residents to discuss and practice patient flow management. Our paper integrates findings from the existing literature to identify a comprehensive list of flow training topics, strategies, and modalities.

### Strengths and limitations

These findings add to a gap in the academic literature regarding the skills ED staff require to improve patient flow and the training strategies and modalities appropriate for delivering flow education. However, the paper also has several limitations. While the data extracted through the integrative review allowed us to identify training topics, strategies, and modalities, it did not provide insight into which topics should be prioritized in a flow curriculum or the advantages or disadvantages of various training approaches. Additionally, since we conducted an integrative review and included a variety of sources, we did not perform a quality assessment of the included articles. Finally, since our objective was to identify a list of relevant training topics, we did not identify specific content that should be taught to develop each skill.

### Clinical implications

Our findings suggest that flow training should be prioritized and standardized in training programs for emergency physicians, nurses, nurse practitioners, and practicing healthcare professionals. Gaining insight into the skills that ED staff require to improve flow and efficiency and the appropriate training strategies and modalities to teach these competencies is critical to enhancing ED flow.

### Research implications

Our findings address steps one and two of Kern’s six-step approach to curriculum development in medical education by identifying the problem and conducting a targeted needs assessment through the integrative review and modified Delphi [16]. Future research is required to complete the subsequent steps in Kern’s model and develop an ED flow training curriculum.

## Conclusion

This paper addresses a significant gap in the academic literature regarding appropriate flow training for ED staff and a practical gap in clinical education for ED physicians, nurses, and nurse practitioners. Our findings suggest that flow training should teach ED staff how to make decisions that improve flow, work more effectively as a team, manage patient backlog and surge, improve leadership skills, and develop situational awareness.

## Supplementary Information

Below is the link to the electronic supplementary material.

Supplementary file1 (DOCX 27 KB)

Supplementary file2 (DOCX 16 KB)

## Footnotes

1 We define training modalities as the method by which certain training strategies are delivered.
