# Supplementary material for: Identifying relevant topics and training methods for emergency department flow training
Source: CJEM. 2022 Oct 15;24(8):837–43. doi: 10.1007/s43678-022-00390-1 (PMC9763133; doi:10.1007/s43678-022-00390-1)
Supplement: Supplementary file 1 — Supplementary file1 (DOCX 27 KB) [file 43678_2022_390_MOESM1_ESM.docx]

| **Table 1: Studies included in the integrative review** | | | | | | | | |
| --- | --- | --- | --- | --- | --- | --- | --- | --- |
| **First author (Year)** | **Title** | **Country** | **Objective** | **Sample**  **Size** | **Design** | **Target Group** | **Training Delivered** | **Focus of the Article** |
| N/A (1998)^12^ | Increase the number of patients you treat in a day: Examine your habits, learn to delegate | N/A | Demonstrate the benefits of ED physicians examining their clinical practice for ways to improve patient flow | N/A | N/A (Commentary) | ED physicians | N/A | Training modality;  Skills and competencies |
| Aaronson, E. (2019)^13^ | Training to Improve Communication Quality: An Efficient Interdisciplinary Experience for Emergency Department Clinicians | United Stated | Create a curriculum for emergency medicine (EM) clinicians to improve provider–patient communication | 298 | Mixed-methods: Survey | ED physicians;  ED Residents;  Nurse practitioners;  Nurses;  Physician assistants | Workshops | Training modality |
| Assid, P. A. (2011)^14^ | Transforming an emergency department: From crisis to excellence | United States | Improve ED processes and efficiency | N/A | Quantitative: Hospital data | Nurses | One on one instruction;  Workshops | Training modality;  Skills and competencies |
| Bandiera, G. (2005)^15^ | Creating effective learning in today’s emergency departments: How accomplished teachers get it done | Canada | Identify effective teaching behaviours among clinical teachers in emergency medicine | 33 | Qualitative: Structured interview survey | N/A | N/A | Training modality |
| Bartlett, J.  (2002)^16^ | The Victorian emergency department collaboration | Australia | Improve clinical wait times and patient satisfaction | 17 hospitals | Observational | ED physicians;  Nurses | Lecture; Workshops | Training modality |
| Bobb, M. R.  (2018)^17^ | Key high-efficiency practices of emergency department providers: A mixed-methods study | United States | Determine the practices that contribute to associated with provider efficiency in community EDs | 35 | Mixed-methods: Interviews; survey | ED physicians; Nurse practitioners; Nurses; Physician assistants | N/A | Training modality; Skills and competencies |
| Bonalumi, N. M. (2017)^18^ | Impact of a planned workflow change: Super track improves quality and service for low-acuity patients at an inner-city hospital | United States | Improve the care provided to low-acuity patients by implementing a Super Track | N/A | Quantitative: Interviews; Hospital data | Not specified (ED staff) | Simulation | Training modality; Simulation |
| Bradt, D. A. (2009)^19^ | Emergency department surge capacity: Recommendations of the Australasian surge strategy working group | Australia | Provide recommendations for clinical management of ED surge | N/A | N/A (Commentary) | N/A | N/A | Skills and competencies |
| Brar, G. (2021)^20^ | Using observation to determine teachable moments within a serious game: A GridlockED as medical education (GAME) study | Canada | Identify which teaching points learners are exposed to while playing the GridlockED game | 32 | Mixed-methods: Survey; Interviews; Observation | ED physicians; ED residents; Nurses | Serious game | Training modality; Skills and competencies; Simulation |
| Chan, T. (2017)^21^ | Failure to flow: An exploration of learning and teaching in busy, multi-patient environments using an interpretive descriptive method | Canada | Identify the best teaching and learning strategies to help emergency physicians (EPs) handle busy, multi-patient ED environments | 20 | Qualitative: Interviews | ED physicians;  ED residents | N/A | Skills and competencies |
| Chan, T. (2018)^22^ | Managing multiplicity: Conceptualizing physician cognition in multipatient environments | Canada | Develop a framework of emergency physician cognition in busy, multi-patient ED environments | 20 | Qualitative: Think aloud exercise | ED physicians; ED residents | Simulation | Training modality;  Skills and competencies;  Simulation |
| Chan, T. (2019)^23^ | Coaching for Chaos: A qualitative study of instructional methods for multipatient management in the emergency department | Canada | Identify the teaching strategies emergency physician teachers employ in overcrowded ED environments and how these strategies are perceived by trainees | 20 | Quantitative: Interviews | ED physicians;  ED residents | One on one instruction; Simulation | Training modality |
| Chan, T. (2020)^24^ | Just the facts: How to teach emergency department flow management | Canada | Summarize approaches to ED flow and management | N/A | N/A (Commentary) | ED residents | N/A | Skills and competencies |
| Chinai, A.  (2018)^25^ | Taking advantage of the teachable moment: A review of learner-centered clinical teaching models | United States | Describe 7 teaching models to optimize learner-centered  teaching in busy clinical settings | N/A | N/A (Commentary) | N/A | Teachable moments | Training modality |
| Chorley, A. (2021)^26^ | Faculty-led opinions of workplace-based methods for graduated managerial teaching (FLOW MGMT): A national cross-sectional survey of Canadian emergency medicine lead educators | Canada | Determine when emergency medicine residents should learn ED management and flow skills | 21 | Quantitative: Survey | ED physicians | N/A | Training modality; Skills and competencies |
| Campbell, S. G. (2004)^27^ | Strategies for managing a busy emergency department | Canada | Suggest flow management strategies | 30 | Quantitative: Survey | ED physicians | N/A | Skills and competencies |
| Craig, S. (2013)^28^ | Registrar in charge shifts’: Learning how to run a busy emergency department | Australia | Assess the impact of a daytime’ registrar in charge’ shift in a tertiary adult ED | 29 | Quantitative: Survey | ED physicians;  ED residents | One-on-one instruction; Rostered didactic sessions | Training modality; Skills and competencies |
| Curran-Smith, J. (2004)^29^ | An experience with an online learning environment to support a change in practice in an emergency department | Canada | Describe experiences with an online training program | N/A | N/A  (Commentary) | Nurses | Online (chat room) | Training modality |
| Egan, H. M. (2021)^30^ | High-efficiency practices of residents in an academic emergency department: A mixed-methods study | United States | Identify actions related to improved efficiency among emergency medicine residents | 35 | Mixed-methods: Interviews; Observation | ED residents | N/A | Skills and competencies |
| Flowerdew, L. (2012)^31^ | Development and validation of a tool to assess emergency physicians’ nontechnical skills | United Kingdom | Develop and validate a tool for assessing nontechnical skills related to safe performance in the ED | 148 ED staff (survey) | Mixed-methods: Survey, interviews, observation | ED physicians;  ED residents;  Nurses | N/A | Skills and competencies |
| Flowerdew, L. (2013)^32^ | A multicentre observational study to evaluate a new tool to assess emergency physicians’ non-technical skills | United Kingdom | Evaluate a new tool to assess emergency physicians’ non-technical skills | 43 | Observational | ED physicians | N/A | Skills and competencies |
| Garg, N. (2014)^33^ | System dynamics as a tool to understand the operations of an emergency department | United States | Describe a game that simulates patient flow in a busy ED | N/A | Simulation | ED physicians | Simulation | Skills and competencies; Simulation |
| Hosking, I. (2018)^34^ | What do emergency physicians in charge do? A qualitative observational study | United Kingdom | Describe the problem solving strategies necessary for the emergency physician in charge | 3 | Qualitative: Interviews; Observation | ED physicians;  Nurses | N/A | Skills and competencies |
| Grant, J. D. (2011)^35^ | Emergency department madness: Tips to maintain sanity and flow | Canada | Describe principles that can be used by emergency physicians to improve efficiency | N/A | N/A (Commentary) | ED physicians | N/A | Skills and competencies |
| Houze-Cerfon, C. H.  (2019)^36^ | Development and evaluation of a virtual research environment to improve quality of care in overcrowded emergency departments: Observational study | France | Develop a virtual ED that replicates patient flow and evaluate its authenticity | 13 | Mixed-methods: Survey, interviews, observation | ED physicians | Simulation | Training modality; Simulation |
| Jonson, C.-O. (2017)^37^ | Short simulation exercises to improve emergency department efficacy for initial disaster management: Controlled before and after study | Sweden | Examine if computer simulation exercises improve head emergency nurses’ self-efficacy and incident management skills | 13 | Quantitative (before and after study): Survey | Nurses | Simulation | Training modality;  Skills and competencies;  Simulation |
| Kelly, S. (2013)^38^ | Improving the ED experience with service excellence focused on teamwork and accountability | United States | Demonstrate that formal ED training in teamwork and accountability can impact the quality of patient care | 26 885 | Quantitative: Survey | ED physicians;  Nurses;  Physician assistants;  Allied health professionals | Workshops; Reading assignments | Training modality;  Skills and competencies |
| Kilner, E. (2010)^39^ | The role of teamwork and communication in the emergency department: A systematic review | Australia | Conduct a systematic review to describe the role of teamwork and communication in the emergency department | N/A | Review | N/A | N/A | Skills and competencies |
| MacIntosh, T. (2019)^40^ | Medical students and metrics: Seven techniques for a win-win situation | United States | Describe techniques to improve ED throughput | N/A | N/A (Commentary) | Medical students | N/A | Training modality |
| McClelland, M. S. (2011)^41^ | The past, present, and future of Urgent Matters: Lessons learned from a decade of emergency department flow improvement | United States | Describe the Urgent Matters program, which aims to improve ED flow | N/A | N/A (Commentary) | N/A | N/A | Skills and competencies |
| Meguerdichian, D. A. (2021)^42^ | Evaluating Nontechnical Skills in US Emergency Departments Using Simulation | United States | Assess the validity and reliability of a United Kingdom emergency medicine assessment tool in the United States | 208 | Mixed-methods: Survey; Observation; Focus Group | ED physicians | N/A | Skills and competencies |
| Nugus, P. (2010)^43^ | The dynamic interaction of quality and efficiency in the emergency department: Squaring the circle? | Australia | Explore the relationship between efficiency and quality of care  in clinical practice | 56 interviews; 12 clinicians observed; 234 hours of semi-structured observations | Qualitative: Interviews; Observation | ED physicians;  Nurses | N/A | Skills and competencies |
| Nugus, P. (2014)^44^ | The emergency department “carousel”: An ethnographically-derived model of the dynamics of patient flow | Australia | Create a conceptual diagram of how emergency clinicians respond to patient flow | 12 | Qualitative: Observation | N/A | N/A | Skills and competencies |
| Sadosty, A. (2008)^45^ | Five simple steps to improve an emergency physician’s efficiency | United States | Present strategies that enable providers to improve their efficiency without changing their style of practice | N/A | N/A (Commentary) | ED physicians | N/A | Skills and competencies |
| Taher, A. (2020)^46^ | Quality improvement initiative for improved patient communication in an ED rapid assessment zone | Canada | Improve patient satisfaction and decrease anxiety through improved communication | 232 patients; 104 clinicians | Mixed-methods: Survey;  Focus groups | ED physicians;  ED residents;  Nurse practitioners;  Nurses;  Medical students;  Physician assistants | N/A | Skills and competencies |
| Tanabe, P. (2008)^47^ | Should you close your waiting room? Addressing ED overcrowding through education and staff-based participatory research | United States | Develop criteria for closing the ED waiting room through staff-based participatory research | 73 | Mixed-methods: Surveys | Nurses | Workshops | Skills and competencies |
| Tanabe, P.  (2009)^48^ | Can education and staff-based participatory research change nursing practice in an era of ED overcrowding? A focus group study | United States | To explore charge nurses’ experiences with adopting close–the–waiting room criteria | 12 | Qualitative: Focus groups | Nurses | N/A | Training modality |
| Tsoy, D. (2019)^49^ | Creating GridlockED: A serious game for teaching about multipatient environments | Canada | Examine how to create a safe teaching environment that exposes junior medical trainees to core ED systems | 32 | Quantitative: Survey | ED physicians;  ED residents;  Nurses | Serious game | Training modality; Simulation |
| Vaillancourt, L. (2018)^50^ | Case study: Gaining physician involvement in quality improvement initiatives: An organizational perspective | Canada | Provide an overview of Lean initiatives from the medical perspective | N/A | Mixed-methods: Interviews; Hospital data | ED physicians | N/A | Skills and competencies |
| van Deen, W. (2019)^51^ | Involving end-users in the design of an audit and feedback intervention in the emergency department setting - A mixed methods study | United States | Evaluate the use of a performance feedback dashboard in reducing length of stay | 71 | Mixed-methods: Survey; Interviews | ED physicians | N/A | Training modality |
| Venugopal, R. (2008)^52^ | A workshop to improve workflow efficiency in emergency medicine | Canada | Determine whether an interactive workshop can improve self-assessed workflow efficiency skills | 46 | Mixed-methods: Survey | ED physicians;  ED residents | Simulation;  Workshops | Training modality; Skills and competencies |
| Walker, A. (2021)^53^ | A simulated scenario to improve resident efficiency in an emergency department | United States | Evaluate senior residents’ comfort level with a multitasking simulation exercise | 6 | Quantitative: Survey | ED residents | Simulation | Skills and competencies; Simulation |
| Wall, O. (2021)^54^ | Teaching acute hospital staff and students about patient flow | Ireland | Examine how patient flow is taught in acute hospitals | 5 | Qualitative: Interviews | Nurses | N/A | Training modality; Skills and competencies |
| Welch, S. J.  (2006)^55^ | Time for a rigorous performance improvement curriculum for emergency medicine residents | United States | Improve the quality of curriculum for emergency medicine residents | N/A | N/A (Commentary) | ED residents | N/A | Skills and competencies |
| Whatley, S. D. (2016)^56^ | Process improvements to reform patient flow in the emergency department | Canada | Discuss the key concepts of ED patient flow, value and efficiency | N/A | Quantitative: Hospital data | N/A | N/A | Skills and competencies |
| Wise, S. (2022)^57^ | A team mental model approach to understanding team effectiveness in an emergency department: A qualitative study | Australia | Demonstrate how the team mental model concept relates to ED team effectiveness | 19 | Qualitative: Interviews | ED physicians;  Nurse practitioners;  Nurses | N/A | Skills and competencies |
