# Supplementary material for: Identifying relevant topics and training methods for emergency department flow training
Source: CJEM. 2022 Oct 15;24(8):837–43. doi: 10.1007/s43678-022-00390-1 (PMC9763133; doi:10.1007/s43678-022-00390-1)
Supplement: Supplementary file 2 — Supplementary file2 (DOCX 16 KB) [file 43678_2022_390_MOESM2_ESM.docx]

**EMBASE (via Embase.com), search conducted 18 January 2022**

| # | **Search Query** | **Results** |
| --- | --- | --- |
| #1 | 'emergency health service'/exp | 117589 |
| #2 | (emergency NEAR/2 (department? OR unit? OR room?)):ab,ti | 29779 |
| #3 | (trauma NEAR/2 (centre? OR center? OR department? OR unit?)):ab,ti | 7265 |
| #4 | (triage NEAR/2 (centre? OR center? OR department? OR unit?)):ab,ti | 121 |
| #5 | 'accident and emergency':ab,ti OR 'accident & emergency':ab,ti OR 'emergency service?':ab,ti | 12010 |
| #6 | #1 OR #2 OR #3 OR #4 OR #5 | 149472 |
| #7 | 'emergency medicine'/exp | 44265 |
| #8 | 'physician'/exp | 852427 |
| #9 | 'physician assistant'/de OR 'advanced practice provider'/de | 10253 |
| #10 | 'emergency nursing'/de | 6878 |
| #11 | 'nurse'/exp | 199350 |
| #12 | 'nursing assistant'/de | 5217 |
| #13 | 'health care personnel'/de OR 'hospital personnel'/exp OR 'medical personnel'/exp | 1264040 |
| #14 | 'rescue personnel'/de | 8498 |
| #15 | 'emergency medical technician?':ab,ti OR paramedic?:ab,ti | 7404 |
| #16 | (emergency NEAR/5 (nurse? OR physician? OR resident? OR clinician? OR provider? OR technician? OR staff OR personnel)):ab,ti | 32132 |
| #17 | (ed NEAR/1 (nurse? OR physician? OR resident? OR clinician? OR provider? OR technician? OR staff OR personnel)):ab,ti | 5514 |
| #18 | #7 OR #8 OR #9 OR #10 OR #11 OR #12 OR #13 OR #14 OR #15 OR #16 OR #17 | 1462439 |
| #19 | 'professional competence'/de | 33248 |
| #20 | skill*:ab,ti OR competenc*:ab,ti OR knowledge:ab,ti | 1324439 |
| #21 | 'continuing education'/de | 32631 |
| #22 | 'in service training'/de | 16611 |
| #23 | 'simulation training'/exp | 7350 |
| #24 | education*:ab,ti OR train*:ab,ti OR instruct*:ab,ti OR learn*:ab,ti | 2042958 |
| #25 | workshop*:ab,ti OR 'work shop*':ab,ti OR webinar?:ab,ti | 61359 |
| #26 | 'interpersonal communication'/exp | 713829 |
| #27 | communication:ab,ti | 335834 |
| #28 | 'teamwork'/de | 19348 |
| #29 | 'team nursing'/de OR 'multidisciplinary team'/de OR 'collaborative care team'/exp | 16795 |
| #30 | teamwork:ab,ti OR 'team work':ab,ti | 17635 |
| #31 | 'interprofessional relation*':ab,ti OR 'inter professional relation*':ab,ti | 561 |
| #32 | 'intraprofessional relations*':ab,ti OR 'intra professional relations*':ab,ti | 39 |
| #33 | 'health personnel attitude'/exp | 198457 |
| #34 | attitude?:ab,ti | 158816 |
| #35 | 'leadership'/de | 78931 |
| #36 | leadership:ab,ti | 51377 |
| #37 | 'assertiveness'/de | 3655 |
| #38 | assertiveness:ab,ti | 2382 |
| #39 | 'awareness'/de | 108301 |
| #40 | 'situation* awareness':ab,ti | 2204 |
| #41 | anticipating:ab,ti | 7250 |
| #42 | 'decision making'/de OR 'clinical decision making'/de | 307337 |
| #43 | decisionmaking:ab,ti OR 'decision making':ab,ti | 217216 |
| #44 | 'time management'/de | 5451 |
| #45 | 'time management':ab,ti OR 'task management':ab,ti OR 'workload management':ab,ti | 3228 |
| #46 | 'task distribution':ab,ti OR 'distribut* tasks':ab,ti | 150 |
| #47 | adherence:ab,ti | 207300 |
| #48 | 'mutual standard maintenance':ab,ti OR 'maintaining standard?':ab,ti | 185 |
| #49 | (provid* NEAR/1 (feedback OR 'feed back')):ab,ti | 8594 |
| #50 | 'workflow'/de | 30013 |
| #51 | 'total quality management'/de | 76490 |
| #52 | 'quality improvement':ab,ti | 65130 |
| #53 | lean:ab,ti | 60062 |
| #54 | 'process improvement':ab,ti | 3617 |
| #55 | 'design thinking':ab,ti | 374 |
| #56 | streaming:ab,ti | 5956 |
| #57 | #19 OR #20 OR #21 OR #22 OR #23 OR #24 OR #25 OR #26 OR #27 OR #28 OR #29 OR #30 OR #31 OR #32 OR #33 OR #34 OR #35 OR #36 OR #37 OR #38 OR #39 OR #40 OR #41 OR #42 OR #43 OR #44 OR #45 OR #46 OR #47 OR #48 OR #49 OR #50 OR #51 OR #52 OR #53 OR #54 OR #55 OR #56 | 4568294 |
| #58 | wait*:ab,ti | 101154 |
| #59 | 'time to treatment'/de | 21352 |
| #60 | ((time OR timeliness) NEAR/2 treatment):ab,ti | 54284 |
| #61 | ((decreas* OR reduce* OR reduction) NEAR/2 time):ab,ti | 97970 |
| #62 | 'response time?':ab,ti | 7699 |
| #63 | 'length of stay'/de | 225969 |
| #64 | 'length of stay':ab,ti OR los:ab,ti | 161737 |
| #65 | 'organizational efficiency'/de | 1182 |
| #66 | efficien*:ab,ti | 1300691 |
| #67 | throughput:ab,ti OR 'through put':ab,ti | 143373 |
| #68 | turnaround:ab,ti OR 'turn around':ab,ti | 13040 |
| #69 | flow:ab,ti | 1043163 |
| #70 | #58 OR #59 OR #60 OR #61 OR #62 OR #63 OR #64 OR #65 OR #66 OR #67 OR #68 OR #69 | 2903809 |
| #71 | #6 AND #18 AND #57 AND #70 | 3604 |
